# Chemically Inhomogeneous RE-Fe-B Permanent Magnets with High Figure of Merit: Solution to Global Rare Earth Criticality

**DOI:** 10.1038/srep32200

**Published:** 2016-08-24

**Authors:** Jiaying Jin, Tianyu Ma, Yujing Zhang, Guohua Bai, Mi Yan

**Affiliations:** 1 School of Materials Science and Engineering, State Key Laboratory of Silicon Materials, Key Laboratory of Novel Materials for Information Technology of Zhejiang Province, Zhejiang University, Hangzhou 310027, China

## Abstract

The global rare earth (RE) criticality, especially for those closely-relied Nd/Pr/Dy/Tb in the 2:14:1-typed permanent magnets (PMs), has triggered tremendous attempts to develop new alternatives. Prospective candidates La/Ce with high abundance, however, cannot provide an equivalent performance due to inferior magnetic properties of (La/Ce)_2_Fe_14_B to Nd_2_Fe_14_B. Here we report high figure-of-merit La/Ce-rich RE-Fe-B PMs, where La/Ce are inhomogeneously distributed among the 2:14:1 phase. The resultant exchange coupling within an individual grain and magnetostatic interactions across grains ensure much superior performance to the La/Ce homogeneously distributed magnet. Maximum energy product (*BH*)_max_ of 42.2 MGOe is achieved even with 36 wt. % La-Ce incorporation. The cost performance, (*BH*)_max_/cost, has been raised by 27.1% compared to a 48.9 MGOe La/Ce-free commercial magnet. The construction of chemical heterogeneity offers recipes to develop commercial-grade PMs using the less risky La/Ce, and also provides a promising solution to the REs availability constraints.

Rare-earths (REs), the natural gems with unique chemical and physical properties, are indispensable components in many emerging and critical technologies^1^,^2^,^3^,^4^,^5^,^6^. In China, the main RE supplier, ~40 wt. % of such strategic sources (Figure S1) are consumed by the 2:14:1-typed Nd-Fe-B magnets^4^, the strongest permanent magnets (PMs) ever invented^7^,^8^,^9^,^10^,^11^,^12^,^13^. However, continually growing demand for Nd-Fe-B magnets and insecure supply for those closely-relied Nd/Pr/Dy/Tb due to geologic scarcity, extraction difficulties and political volatility has caused severe concerns about REs availability, which may exert spill-over effects on global economy, and security in the case of defense or military sectors^2^,^6^. Meanwhile, the large backlog of highly-abundant and inexpensive La/Ce (by-products of Nd/Pr/Dy/Tb during mineral extraction) due to the inferior magnetic properties of (La/Ce)_2_Fe_14_B to Nd_2_Fe_14_B^11^,^12^,^13^,^14^,^15^,^16^,^17^,^18^,^19^,^20^,^21^,^22^ is a longstanding bottleneck for balanced utilization of RE resources^4^,^13^,^23^.

The figures of merit, (*BH*)_max_, *B*_r_, especially *H*_cj_, for the domain-reversal controlled Nd-Fe-B magnets are highly sensitive to the intrinsic magnetic properties of 2:14:1 phase and the structural inhomogeneities. The locally low magnetocrystalline anisotropy close to the defects as well as the short-range exchange couplings between un-isolated hard phase grains lead to lower coercivity than the theoretical *H*_A_, known as Brown’s paradox^24^. Basic approach to achieve high macroscopic performance is to eliminate the structural defects^24^, e.g. refining grain size^25^, strengthening the local anisotropy at transition regions^26^ and constructing continuous grain boundary (GB) layers to decouple adjacent grains^27^. Note that the above principles are relying on that the chemistry of 2:14:1 matrix phase is homogeneous either within an individual grain or among all the grains, despite a slight deviation at the interface between GBs and main phase. Early work on (Nd, Ce/La)_2_Fe_14_B based magnets has demonstrated that homogeneous La/Ce substitution for Nd/Pr deteriorates magnetic properties to disappointingly low values^14^,^15^,^16^ due to inferior intrinsic properties of La_2_Fe_14_B and Ce_2_Fe_14_B to Nd_2_Fe_14_B (the room temperature saturation magnetic polarization *J*_S_ and anisotropy field *H*_A_ for La_2_Fe_14_B/Ce_2_Fe_14_B/Nd_2_Fe_14_B compounds are 1.38/1.17/1.60 T, and 20/26/73 kOe, respectively^18^). Even when the structural inhomogeneities are reduced through optimizing composition design and manufacturing condition, only 5 wt. % Ce is allowed to substitute for Nd when preparing (*BH*)_max_ ~ 40 MGOe sintered magnet^14^. Consequently, it poses a big challenge for the (Nd, Ce/La)-Fe-B magnets to provide an equivalent weight-bearing capacity or performance, compared to the un-doped Nd-Fe-B host.

In this work, we report a new prototype of La/Ce-rich RE-Fe-B PMs with chemical heterogeneity, which was constructed by mixing the La/Ce-free and La/Ce-rich RE_2_Fe_14_B terminal powders, followed by compacting/alignment, sintering and post-annealing procedures. The resultant short-range exchange couplings of local regions within an individual 2:14:1 main phase grain, and the long-range magnetostatic interactions among different 2:14:1-typed grains in these magnets facilitate superior magnetic performance to those prepared by directly alloying at the same average composition (Table S1). It offers a new approach to fabricate RE-Fe-B PMs with high cost performance, and also provides a promising solution to the global RE criticality.

## Results

**Figure 1:**
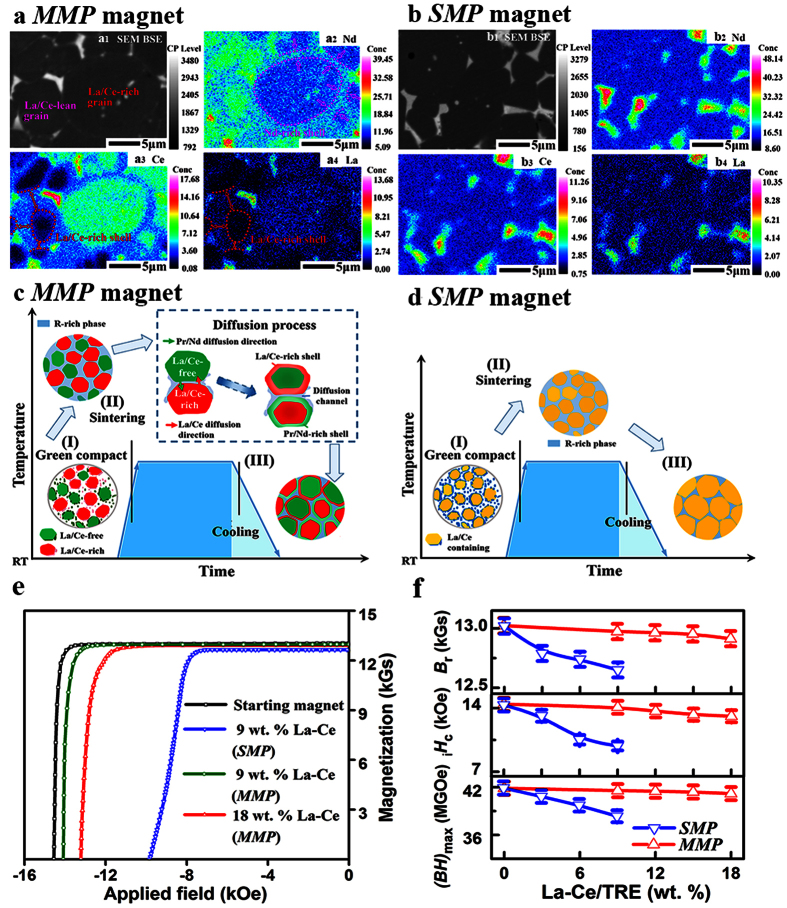
Chemical heterogeneity and magnetic performance of *MMP* magnets compared to the chemically homogeneous *SMP* ones. BSE-SEM micrographs (bright and grey contrasts refer to RE-rich and matrix phases, respectively) and corresponding elemental mappings of Nd, Ce and La for (**a**) *MMP* magnet with 18 wt. % La-Ce/TRE and (**b**) *SMP* magnet with 9 wt. % La-Ce/TRE. Schematics in (**c**,**d**) show the evolution of different microstructures for *MMP* and *SMP* magnets, respectively. (**e**) Room temperature demagnetization curves for *SMP* and *MMP* magnets. (**f**) Dependence of magnetic properties on La-Ce concentration for *SMP* and *MMP* magnets.

Despite that the back-scattered SEM image (Fig. 1a1) reveals a bi-phase morphology, the grey main phase and bright RE-rich phase, the electron probe microanalyzer (EPMA) (Fig. 1a2–a4) depicts that RE concentration in the main phase differs a lot either over local regions within an individual grain or across grains, being completely different from the directly alloyed magnet where La/Ce/Nd are homogenously distributed in the main phase (Fig. 1b). Within one individual grain, gradient RE distributions are formed, i.e. obvious core-shell structures that the La/Ce-rich grain is surrounded by a Nd-rich shell, and the La/Ce-lean one with a La/Ce-rich shell simultaneously. The coexistence of La/Ce-lean and La/Ce-rich grains reveals a global RE concentration fluctuation. Such peculiar chemical heterogeneity originates from the initial composition gradient between La/Ce-free (green) and La/Ce-rich (red) 2:14:1 terminals (detailed compositions listed in Table S1) as schematically shown in Fig. 1c. During liquid-phase sintering, elemental interdiffusion is generated via the liquid RE-rich phase, e.g. La/Ce diffuse from La/Ce-rich grains to La/Ce-free ones (red arrow), and Pr/Nd follows the reverse direction (green arrow). However, on account of limited thermal diffusion below the melting points of RE_2_Fe_14_B compounds, La/Ce and Pr/Nd cannot immigrate homogenously, leading to the global chemical fluctuations. Meanwhile, for a specific grain, the high-temperature diffusion state is retained, leading to the aforementioned local chemical heterogeneity (core-shell structure). Consequently, such chemically inhomogeneous magnets contain multiple main phases (hereafter, denoted as *MMP* magnet), which are different from the single main phase (*SMP*) structure in a chemically homogeneous magnet (Fig. 1b,d).

Room-temperature demagnetization curves in Fig. 1e show that (*BH*)_max_, *H*_cj_ and *B*_r_ for *MMP* magnets are all superior to the *SMP* ones. Compared to the La/Ce-free starting magnet (*B*_r_ = 13.02 kGs, *H*_cj_ = 14.4 kOe and (*BH*)_max_ = 41.9 MGOe), *SMP* magnets suffer sharp deteriorations in magnetic performance, consistent with previous work that both *J*_S_ and *H*_A_ decay when La/Ce homogeneously substitutes for Nd in the 2:14:1 phase^18^. With 9 wt. % La-Ce, *H*_cj_ decreases drastically to 9.8 kOe by −31.9%, accompanied with significant falls in *B*_r_ to 12.65 kGs and (*BH*)_max_ to 38.3 MGOe. In contrast, for the *MMP* magnet with 9 wt. % La-Ce, (*BH*)_max_ reaches 41.6 MGOe, and *H*_cj_ is 14.0 kOe, which are 8.6% and 42.9% higher than those of *SMP* magnet, respectively. Further increasing La-Ce content to 18 wt. %, magnetic properties of the *MMP* magnet are even superior to those of the *SMP* one with only 9 wt. % La-Ce. Such superiority can be observed over a quite wide composition window (Fig. 1f). More importantly, the chemical heterogeneity does not produce obvious kinks or steps in the demagnetization curves, whose shapes are fairly identical to the commercial-grade Nd-Fe-B magnets. The high squareness factor^28^ (>95%) is a persuasive indicator for the existence of intergrain interactions in these *MMP* magnets. When measuring the magnetic performance up to elevated temperatures, *MMP* magnets also exhibit better thermal stabilities than *SMP* ones (Figure S2).

**Figure 2:**
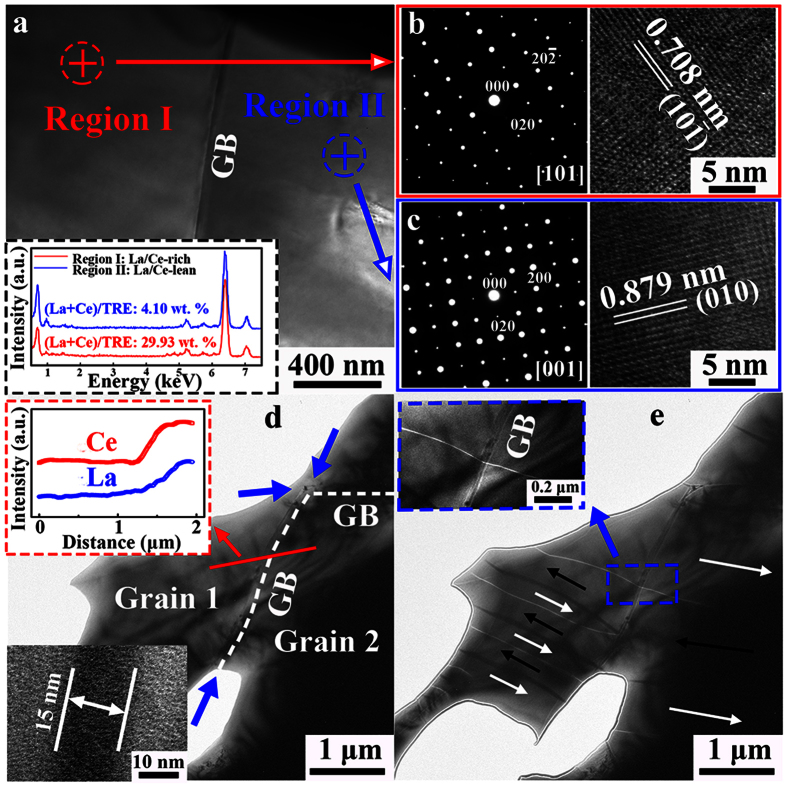
Local crystal and domain features of *MMP* magnet with 18 wt. % La-Ce. (**a**) Bright field image. The inset is EDS spectra at regions I and II, showing the detailed (La + Ce)/TRE ratio (wt. %). (**b**) SAED pattern and HRTEM image of region I in (a). (**c**) SAED pattern and HRTEM image of region II in (a). (**d**) In-focus and (**e**) over-focus Lorentz TEM images of the thermally demagnetized sample (*c*-axis in-plane view). In the in-focus image, neighboring grains 1 (low La/Ce) and 2 (high La/Ce) are separated by the GB layers (indicated by white dotted curves and blue arrows). The upper inset shows the EDS line scan (red solid line), and the bottom one is an enlarged view of GB regions (~15 nm). The over-focus image reveals bright and dark contrasts of the domain walls (an alternating 180° wall pattern, as depicted by arrows), which are produced by opposite magnetizations between adjacent domains. The inset in (**e**) enlarges a continuous domain wall across the thick GB.

TEM characterizations of *MMP* magnets show that inhomogeneous La/Ce distribution within or across the grains does not break the crystal symmetry of the main phase (Fig. 2 and Figure S3), but leads to dramatically differed lattice parameters. Elemental detections at different grains (inset of Fig. 2a) reveal that the ratio of La-Ce/TRE varies, i.e. ~29.93 wt. % in region I, close to the La/Ce-rich terminal, and ~4.10 wt. % (La/Ce-lean) in region II. Selected area electron diffraction (SAED) patterns (Fig. 2b,c) demonstrate that both regions possess the same tetragonal crystal symmetry (space group *P*4_2_/*mnm*^18^). Their lattice parameters, however, are rather different. In region I, the *d*-spacing is 0.708 nm for the 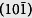 plane, fairly lower than the standard JCPDS data (0.714 nm) for Nd_2_Fe_14_B^18^. In region II, the *d*-spacing is 0.879 nm for (010) plane, close to 0.880 nm of JCPDS data. As La-Ce substitution in the 2:14:1 phase basically leads to a contracted unit cell^16^, the higher La-Ce content, the larger deviation of *d*-spacing from Nd_2_Fe_14_B. Similarly, within one individual main phase grain, there also exists local regions with different La-Ce contents, possessing the same tetragonal crystal symmetry (Figure S3). As a result, the intrinsic magnetic properties, i.e. *J*_S_ and *H*_A_, of the 2:14:1 tetragonal phase with different La/Ce contents have strong local variations accordingly.

A following critical question then arises: how does the local fluctuation of intrinsic magnetic properties influence the macroscopic magnetization reversal? To answer this question, local magnetic domain structure of the *MMP* magnet is characterized by Lorentz TEM (Fig. 2d,e). The domain width *δ* is fairly uneven both within one individual grain and adjacent grains. In grain 1, *δ* is larger in the upper part than that of the bottom one. *δ* for grain 2 is almost twice the width for the neighboring grain 1. The non-equidistant domain width reflects different intrinsic magnetic properties in local regions led by the chemical heterogeneity and short-range exchange coupling effects. Besides, some domain walls are continuous (see the inset of Fig. 2e) across the relatively thick GB (~15 nm), suggesting that the GB layer cannot isolate completely the adjacent grains. Such connecting intergrain domains have been previously ascribed to exchange coupling or magnetostatic interaction^29^. However, since the adjacent grains are in micron size and separated by thick GB layers (inset of Fig. 2d), the intergrain exchange coupling could be very weak and the magnetostatic interaction dominates. The large domains produced by magnetic interactions are also observed using Kerr microscopy (Figures S4 and S5).

**Figure 3:**
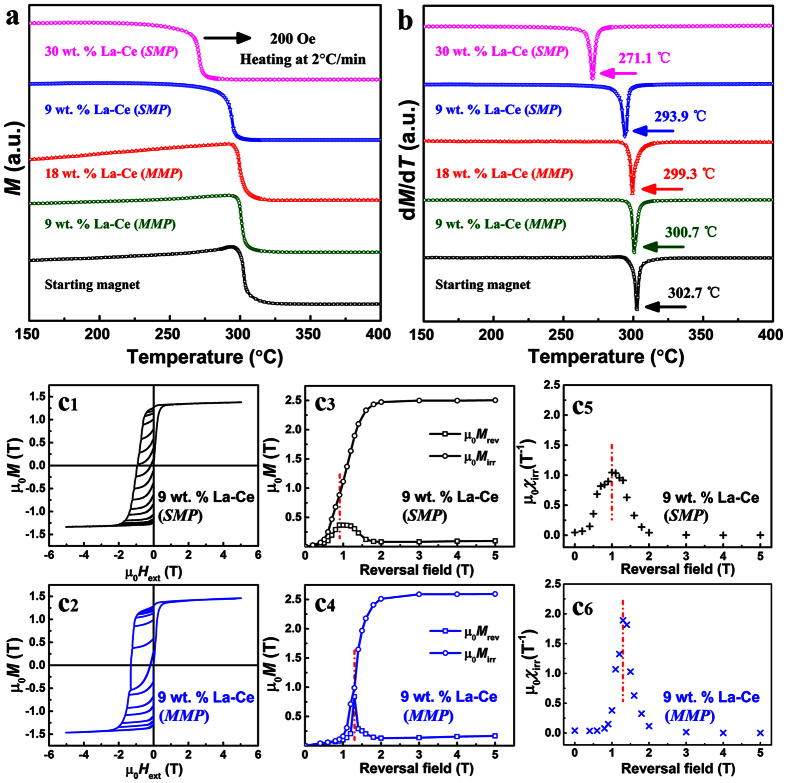
Short-range and long-range magnetic interactions. (**a**) *M* - *T* and (**b**) d*M*/d*T* - *T* curves for *SMP* and *MMP* magnets upon heating at 2 °C/min under 200 Oe. Initial magnetization curves and recoil loops on the demagnetization curve for 9 wt. % La-Ce containing (c_1_) *SMP* and (c_2_) *MMP* magnets. The corresponding dependences of *μ*_0_*M*_irr_ and *μ*_0_*M*_rev_ for *SMP* and *MMP* magnets on the applied reversal field are shown in (c_3_) and (c_4_), respectively. Calculated *μ*_0_*χ*_irr_ as a function of reversal field for *SMP* and *MMP* magnets are given in (c_5_) and (c_6_), respectively.

Further magnetic measurements (Fig. 3) demonstrate the co-existence of short-range and long-range interactions in *MMP* magnets. The thermo-magnetic measurements (Fig. 3a and b) reveal different Curie temperatures (*T*_C_) between *SMP* and *MMP* magnets with the same average composition. Since *T*_C_ for La_2_Fe_14_B/Ce_2_Fe_14_B (257/151 °C) is lower than those of Nd_2_Fe_14_B/Pr_2_Fe_14_B/Gd_2_Fe_14_B (312/292/388 °C)^18^, *SMP* magnet with 9 wt. % La-Ce exhibits a drastic decay to 293.9 °C. Surprisingly, only a slight decline is observed for the *MMP* magnets, with *T*_C_ of 300.7 °C for 9 wt. % La-Ce and 299.3 °C for 18 wt. % La-Ce, respectively. As *T*_C_ is controlled by 3d-3d and RE-Fe exchanges^19^,^30^,^31^, and La/Ce substitution for Pr/Nd in the 2:14:1 lattice decrease the de Gennes factor, higher *T*_C_ of *MMP* magnets than the *SMP* one with the same La-Ce concentration (9 wt. % La-Ce) may reflect strengthened short-range exchange interactions against thermal perturbation in chemically inhomogeneous *MMP* magnets (further explanation provided in the Supplementary Information). The possible influences of magnetic impurities such as α-Fe (*T*_C_ ~ 780 °C^32^), Fe_2_B (*T*_C_ ~ 740 °C^32^), CeFe_2_ (*T*_C_ ~ −38 °C^12^) are excluded as their Curie transitions occur at totally different temperature zones from that of 2:14:1 phase. Moreover, these impurities are not detected by the Rietveld analysis of X-ray diffraction (XRD) patterns (Figures S6 and S7). Reversible and irreversible magnetization *μ*_0_*M*_rev_ and *μ*_0_*M*_irr_, and irreversible susceptibility *μ*_0_*χ*_irr_ of the recoil loops are plotted in Fig. 3c, following refs 28 and 33. The *MMP* magnet possesses a much larger magnetization at 5 T than that of the *SMP* one at the same average composition. Maximum *μ*_0_*M*_rev_ is as large as ~0.7 T for *MMP* magnet, twice of ~0.35 T for the *SMP* one with the same average composition, suggesting stronger exchange-coupling within the former^33^. Nevertheless, only one peak is observed in *μ*_0_*χ*_irr_ at the nucleation field (*H*_N_) of ~1.4 T, indicating a collective magnetization reversal mechanism for the *MMP* magnet. It further manifests the essentially single/homogenous hard magnetic phase behavior, supporting the high squareness factor in Fig. 1e. Moreover, in comparison with the *SMP* magnet, the switching field distribution (SFD)^28^,^33^,^34^ for the *MMP* sample is much narrowed, suggesting a more uniform magnetization reversal behavior. Consequently, single peak in *μ*_0_*χ*_irr_, concentrated SFD, complemented with high squareness of demagnetization curves, unveil that magnetostatic interaction plays a dominant role on the “uniform” magnetization reversal of the *MMP* magnets.

## Discussion

Here we report a novel prototype of La/Ce-rich RE-Fe-B PMs with chemical heterogeneity either over local regions within an individual grain or across grains, being completely distinct from the *SMP* magnet with REs homogenously distributed in the main phase (Fig. 1b), and those with singular core-shell structure (Dy-enriched^35^,^36^,^37^ or La/Ce-enriched outer layer^12^) as well. Besides the peculiar chemical heterogeneity of *MMP* magnet, its high magnetic performance further underscores the dependences of local intrinsic magnetic property/lattice parameter on the composition and its close ties to the extrinsic magnetic properties. It is remarkable that the short-range exchange coupling and long-range magnetostatic interaction can induce higher remanence and coercivity simultaneously than those of *SMP* magnets with the same average composition.

The above findings may provide new insights into the magnetization reversal mechanism in PMs. Within one single 2:14:1 main phase grain, there exists local regions with varied La/Ce contents and intrinsic magnetic properties accordingly, e.g. *J*_S_ and *H*_A_. The La/Ce-rich local region (magnetically softer with lower *J*_S_ and *H*_A_) exchange-coupled with the La/Ce-lean one (magnetically harder with higher *J*_S_ and *H*_A_) inside an individual grain, being beneficial to the magnetic properties, especially for *B*_r_ and (*BH*)_max_. For instance, with the same La-Ce content of 9 wt. %, *B*_r_ for the *MMP* magnet is substantially higher than that of *SMP* one. A special feature for the *MMP* magnet is that adjacent local regions within one 2:14:1 matrix grain possess the same crystal structure (tetragonal) and orientation, and there is no phase boundary or interface layer (only small lattice distortion exists) deteriorating their exchange coupling effect. In addition, the magnetostatic interactions across grains at microscale not only induce “harder” magnetization but also impede the magnetization reversals in *MMP* magnets (Figures S4 and S5). Once reverse domains nucleate in deteriorated regions (close to GB), the neighboring regions/grains with higher *H*_A_ impedes its quick immigration. Consequently, it takes a relatively long progress to achieve complete domain reversals among the whole magnet, thereby generating a much larger coercivity than *SMP* ones. It should be addressed that in *SMP* magnets, magnetostatic interactions^38^ also exist between 2:14:1 phase grains with different alignments. Such interactions may enhance the coercivity, but are not favorable to obtain high remanence. By the means of constructing chemical heterogeneity both within and across 2:14:1 main grains, the bottleneck concern that high La/Ce concentration are not allowed in fabricating commercial Nd-Fe-B PMs can be solved.

**Figure 4:**
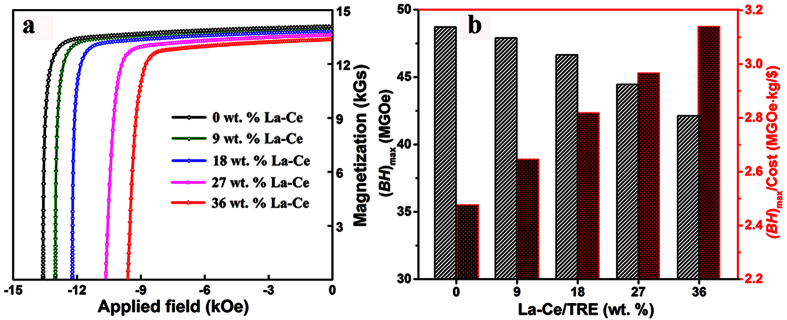
Performance for proof-of-principle *MMP* magnets. (**a**) Demagnetization curves and (**b**) (*BH*)_max_ (black) and cost performance (red) versus La-Ce content for *MMP* magnets.

The chemical heterogeneity also affords a rich spectrum of possibilities for further improving the magnetic performance. On one hand, the final magnetic performance of *MMP* magnets is highly dependent on the La/Ce-free and La/Ce-rich terminals. To increase the La-Ce concentration in the final *MMP* magnets, a higher La-Ce/TRE ratio in the La/Ce-rich terminal is a necessity, which unfortunately may result in different physical/chemical characteristics of both the 2:14:1 matrix phase and RE-rich phase, and the formation of impurity phases^12^, deteriorating the magnetic performance (Table S1). In the present work, we chose another La/Ce-free (Nd, Pr)-Fe-B starting magnet with (*BH*)_max_ of 48.9 MGOe, and a La/Ce-rich terminal with 50 wt.% La-Ce/TRE, to prepare proof-of-principle *MMP* magnets. A high (*BH*)_max_ of 42.2 MGOe can be obtained even when the La-Ce content is as high as 36 wt. % (see Fig. 4 and Table S1). The cost performance, defined as (*BH*)_max_/cost, is ~3.14 MGOe·kg/$, which is 1.27 times of the starting magnet (~2.47 MGOe·kg/$). More comparisons with other PMs are displayed in Figure S8. On the other hand, substantial enhancements in both remanence and coercivity of *MMP* magnets can be expected by modified multi-phase morphologies, i.e. to optimize the sintering and annealing procedures. It highlights future tasks on tuning the chemical heterogeneity and the microstructure to further improve the cost performance.

In summary, efforts to find substitutes for Nd-Fe-B PMs have been under way for 20 years, but to little avail. In our work, however, a promising solution to the tightening global REs criticality is unveiled by preparing high cost performance RE_2_Fe_14_B magnets with large La-Ce concentration. The hallmark lies in the chemical heterogeneity within/across the magnetically hard 2:14:1 phase grains, making it one crucial topic from both the fundamental and application points of view. Theoretically, it offers a rich field for studying the interplay between chemical heterogeneity, interaction at different scales and extrinsic magnetic properties in hard magnetic materials. Technologically, since most procedures remain the same as the conventional approach, the construction of chemical heterogeneity is promising for manufacturing La-Ce containing RE_2_Fe_14_B PMs in mass production, which may greatly promote the sustainability, balance and diversity of global RE industry.

## Methods

### Magnet preparation

Magnetic powders with nominal compositions of (Pr, Nd)_29.8_Gd_1.7_Fe_bal_*M*_1.1_B_1.0_ (I: La/Ce-free terminal) and (Pr, Nd)_20.3_(La, Ce)_9.5_Gd_1.7_Fe_bal_*M*_1.1_B_1.0_ (II: La/Ce-rich terminal, with 30 wt. % La-Ce concentration of TRE) (*M* = Cu, Al, Nb, Zr, in wt. %) were prepared by induction melting, strip casting, hydrogen decrepitation and jet milling. The raw material Pr-Nd alloy denotes a 20 wt. % Pr-80 wt. % Nd composition, and La-Ce alloy is composed of 35 wt. % La and 65 wt. % Ce. By tuning the mass ratios of the two typed powders (see Table S1), *MMP* magnets of 9, 12, 15, 18 wt. % La-Ce (La-Ce content of TRE) were prepared via conventional powder metallurgy process. After pressed under 5.5 MPa in a perpendicular magnetic field of 1.5 T and isostatic compressing under 200 MPa, the green compacts were sintered at 1000 ~ 1075 °C and subsequently annealed at 870 ~ 900 °C and 460 ~ 620 °C. Meanwhile, the starting magnet (by only using La/Ce-free terminal) and *SMP* magnets (by direct alloying 3, 6, 9 wt. % La-Ce into component I) were prepared under the same condition. XRD patterns (10° ≤ 2θ ≤ 100° with a step of 0.02° and a counting time of 4s per step using SHIMADZU XRD-6000) with Rietveld refinement (Rietica software) confirm that the La/Ce-free and La/Ce-rich terminal, *SMP* and *MMP* magnets possess the similar phase components, consisting of matrix RE_2_Fe_14_B phase and minor RE-rich phase (Figures S6 and S7). Calculated from this average measurement, it follows well that the higher La-Ce content, the smaller lattice parameters *a* and *c* for 2:14:1 phase.

### Measurements and characterizations

Elemental concentration mapping was performed using an electron probe microanalyzer (EPMA) with wavelength dispersive X-ray spectrometer (WDS). Magnetic properties were measured by an AMT-4 magnetometer. Curie temperature was determined via measuring the thermomagnetic curve upon heating to 400 °C at 2 °C/min with an external field of 200 Oe. Initial magnetization curve and recoil loops were characterized by a vibrating sample magnetometer (VSM) up to 5 T. The thermal stability was evaluated by temperature coefficients of remanence (*α*) and coercivity (*β*) (from 20 to 100 °C), and the irreversible loss of the open-circuit flux (from 20 to 150 °C). Microstructure and electron diffraction were characterized using the transmission electron microscope (JEM-2100F) equipped with EDS. The magnetic domain structure was observed using Lorenz TEM (Fresnel method) and digitally enhanced Kerr microscope. Samples for TEM characterization were prepared by standard mechanical grinding, dimpling (Gatan 656) and ion milling (Gatan 691). Ion-beam thinning was carried out on both sides of the specimen at an inclination angle of 8° between the beam and the specimen surface. Samples for Kerr-image observations were prepared by standard grinding and polishing.

## Additional Information

**How to cite this article**: Jin, J. *et al.* Chemically Inhomogeneous RE-Fe-B Permanent Magnets with High Figure of Merit: Solution to Global Rare Earth Criticality. *Sci. Rep.* **6**, 32200; doi: 10.1038/srep32200 (2016).

## Supplementary Material

Supplementary Information
