# Supplementary material for: Chemically Inhomogeneous RE-Fe-B Permanent Magnets with High Figure of Merit: Solution to Global Rare Earth Criticality
Source: Sci Rep. 2016 Aug 24;6:32200. doi: 10.1038/srep32200 (PMC4995380; doi:10.1038/srep32200)
Supplement: Supplementary Information [file srep32200-s1.pdf]

Supplementary Information

Chemically Inhomogeneous RE-Fe-B Permanent Magnets with High Figure of Merit: Solution to Global Rare Earth Criticality

Jiaying Jin, Tianyu Ma*, Yujing Zhang, Guohua Bai & Mi Yan*

School of Materials Science and Engineering, State Key Laboratory of Silicon Materials, Key Laboratory of Novel Materials for Information Technology of Zhejiang Province, Zhejiang University, Hangzhou 310027, China

E-mail: maty@zju.edu.cn; mse_yanmi@zju.edu.cn

Table S1. Magnet compositions and performance. La-Ce/TRE, mass ratio of La/Ce-free terminal I/(I+II), average compositions, magnetic and cost performance (groups 1 and 2 are using 41.9 MGOe and 48.9 MGOe commercial magnets as the La/Ce-free terminal, respectively). Ter. I denotes the La/Ce-free terminal, Ter. II denotes the La/Ce-rich one. Data in Figures 1-3 are from group 1, and data in Figure 4 are from group 2. Data of Ref. 12 are listed for comparison.

Specimens		La-Ce /TRE (wt. %)	I /(I+II) (wt. %)	Average composition (wt. %)	$(BH)_{max}$ (MGOe)	B_r (kGs)	H_{cj} (kOe)	Cost performance (MGOe·kg/$)	T_C (°C)
Group 1	Ter.I	0	100	$(Pr,Nd)_{29.8}Gd_{1.7}Fe_{bal}M_{1.1}B_{1.0}$	41.9	13.02	14.4	2.42	302.7
	Ter.II	30	0	$(La,Ce)_{9.5}(Pr,Nd)_{20.3}Gd_{1.7}Fe_{bal}M_{1.1}B_{1.0}$	/	/	/	/	271.1
	SMP	3	/	$(La,Ce)_{1.0}(Pr,Nd)_{28.8}Gd_{1.7}Fe_{bal}M_{1.1}B_{1.0}$	40.8	12.79	13.2	2.43	/
		6	/	$(La,Ce)_{1.9}(Pr,Nd)_{27.9}Gd_{1.7}Fe_{bal}M_{1.1}B_{1.0}$	39.7	12.74	10.5	2.43	/
		9	/	$(La,Ce)_{2.8}(Pr,Nd)_{26.7}Gd_{1.7}Fe_{bal}M_{1.1}B_{1.0}$	38.3	12.65	9.8	2.41	293.9
	MMP	9	70	$(La,Ce)_{2.8}(Pr,Nd)_{26.7}Gd_{1.7}Fe_{bal}M_{1.1}B_{1.0}$	41.6	12.97	14.0	2.62	300.7
		12	60	$(La,Ce)_{3.8}(Pr,Nd)_{26.0}Gd_{1.7}Fe_{bal}M_{1.1}B_{1.0}$	41.5	12.96	13.6	2.70	/
		15	50	$(La,Ce)_{4.8}(Pr,Nd)_{25.0}Gd_{1.7}Fe_{bal}M_{1.1}B_{1.0}$	41.4	12.95	13.2	2.78	/
		18	40	$(La,Ce)_{5.7}(Pr,Nd)_{24.1}Gd_{1.7}Fe_{bal}M_{1.1}B_{1.0}$	41.2	12.91	13.0	2.86	299.3
Group 2	Ter.I	0	100	$(Pr,Nd)_{30.5}Fe_{bal}M_{1.4}B_{1.0}$	48.9	14.09	13.6	2.47	/
	Ter.II	50	0	$(La,Ce)_{15.3}(Pr,Nd)_{15.2}Fe_{bal}M_{1.4}B_{1.0}$	/	/	/	/	/
	MMP	9	82	$(La,Ce)_{2.7}(Pr,Nd)_{27.8}Fe_{bal}M_{1.4}B_{1.0}$	47.9	14.03	13.0	2.65	/
		18	64	$(La,Ce)_{5.5}(Pr,Nd)_{25.0}Fe_{bal}M_{1.4}B_{1.0}$	46.7	13.84	12.2	2.82	/
		27	46	$(La,Ce)_{8.2}(Pr,Nd)_{22.3}Fe_{bal}M_{1.4}B_{1.0}$	44.5	13.63	10.7	2.97	/
		36	28	$(La,Ce)_{11.0}(Pr,Nd)_{19.5}Fe_{bal}M_{1.4}B_{1.0}$	42.2	13.38	9.6	3.14	/
Ref 12	Ter.I	0	100	$(Pr,Nd)_{28.8}Dy_{1.8}Fe_{bal}M_{1.4}B_{1.0}$	42.4	13.29	16.9	/	322
	Ter.II	78.5	0	$(La,Ce)_{25.6}(Pr,Nd)_{7.0}Fe_{bal}M_{0.6}B_{1.0}$	2.4	9.475	0.5	/	243
	Magnet	16.2	20	$(La,Ce)_{5.0}(Pr,Nd)_{24.5}Dy_{1.4}Fe_{bal}M_{1.2}B_{1.0}$	34.0	12.09	10.7	/	309

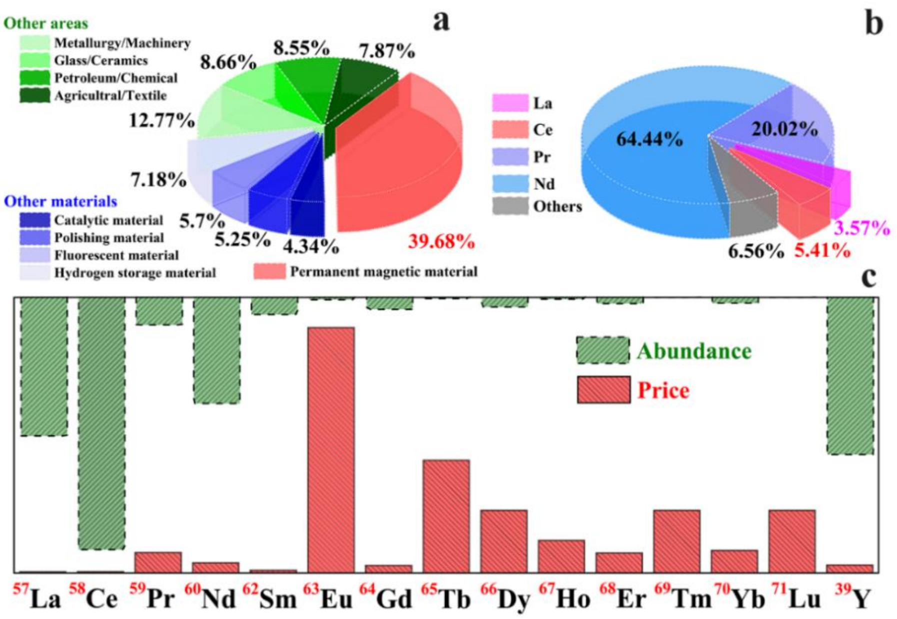

Figure S1. Current utilizations of rare earths in China. (a) RE utilizations in different fields. Permanent magnetic material is the most important application field, consuming 39.68% of the total RE elements. (b) Utilizations of different RE elements in permanent magnets. Only a limited proportion of Ce (5.41 %) and La (3.57 %) are applied in this field. (c) Abundance and price (as of December 2015) of different RE elements.

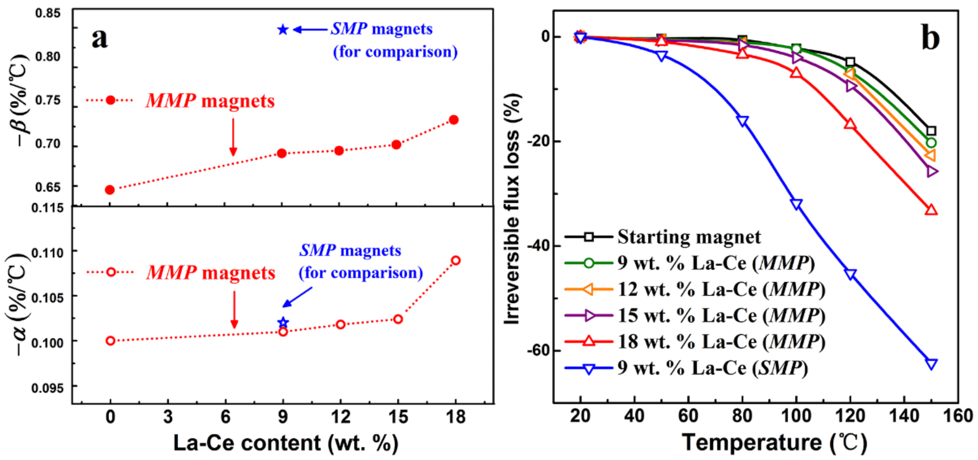

Figure S2. Thermal stability of *SMP* and *MMP* magnets. (a) Temperature coefficients of remanence α and coercivity β (from 20 to 100 °C). (b) Dependence of irreversible flux loss on the temperature (from 20 to 150 °C) for *SMP* and *MMP* magnets.

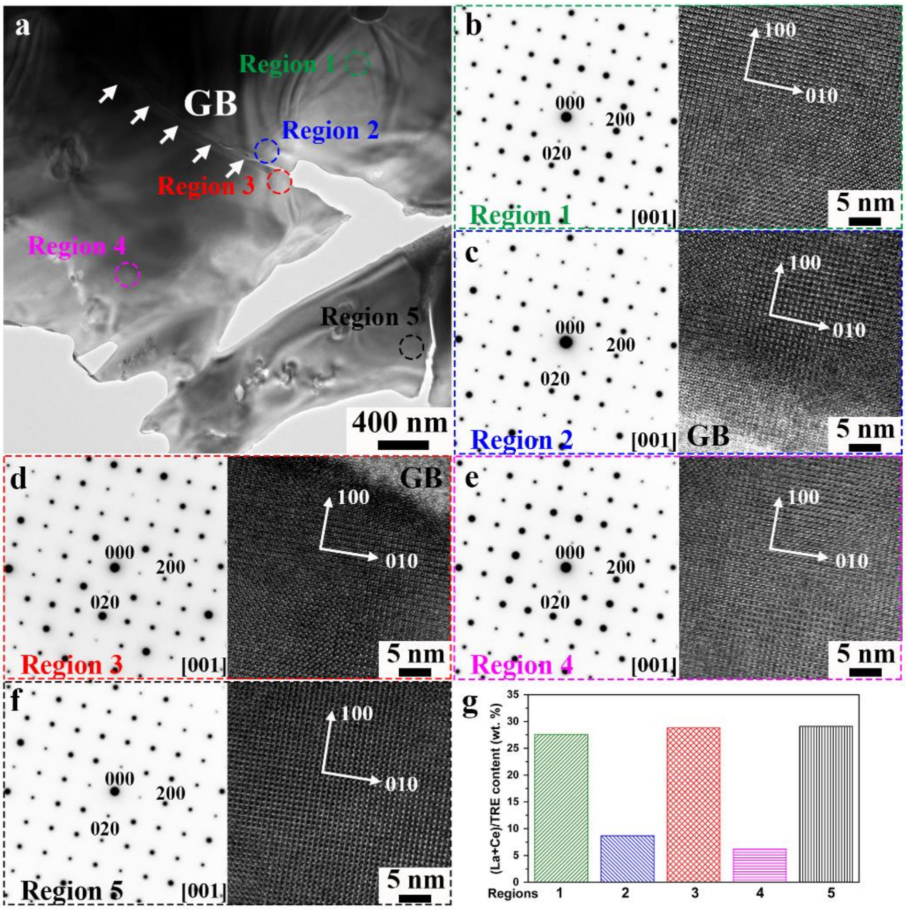

Figure S3. Local crystal features of the *MMP* magnet. (a) Bright field image showing five local regions numbered 1~5. Their corresponding SAED patterns and HRTEM images are shown in (b) to (f), respectively. Their (La+Ce)/TRE content (wt. %) are plotted in (g).

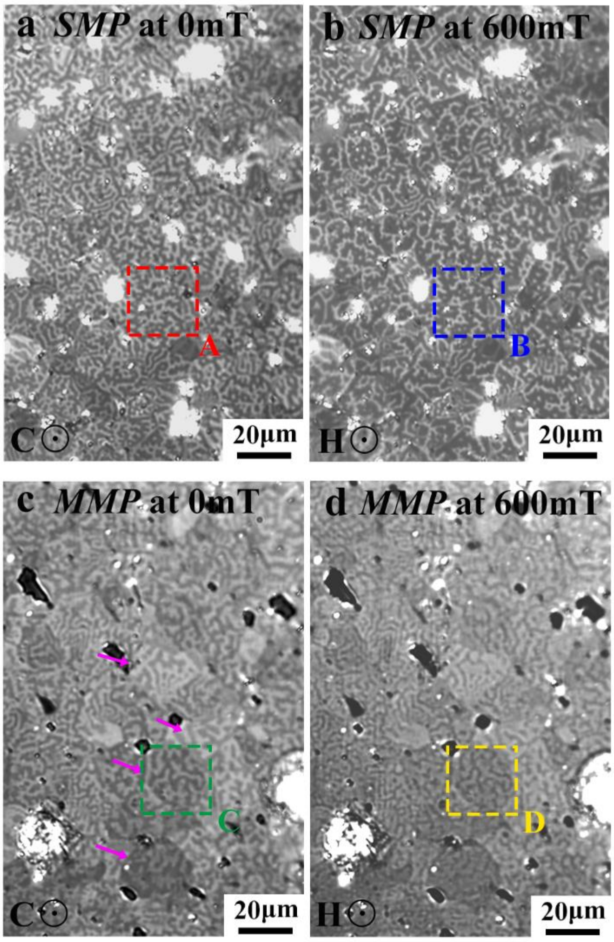

Figure S4. ***C-axis* out-of-plane Kerr view of *SMP* and *MMP* magnets.** Magneto-optical Kerr images of thermally demagnetized (a) *SMP* magnet with 9 wt. % La-Ce and (c) *MMP* magnet with 18 wt. % La-Ce. Average domain width in *MMP* magnet is almost twice the size of the *SMP* one. The pink arrows in (c) depicts the domain clusters that joint together with ~20 μm wide, and are obviously larger than the average grain size (~6.5 μm), indicating a strong exchange coupling within the 2:14:1 grains and long-range magnetostatic interactions in *MMP* magnets. The corresponding domain patterns under 600 mT (parallel to *c*-axis) are shown in (b) and (d). The intersecting domain group changes barely, suggesting a higher stability of the *MMP* magnet in preserving its virgin domain patterns than the *SMP* one. The box areas A, B, C and D are pointed out for comparisons.

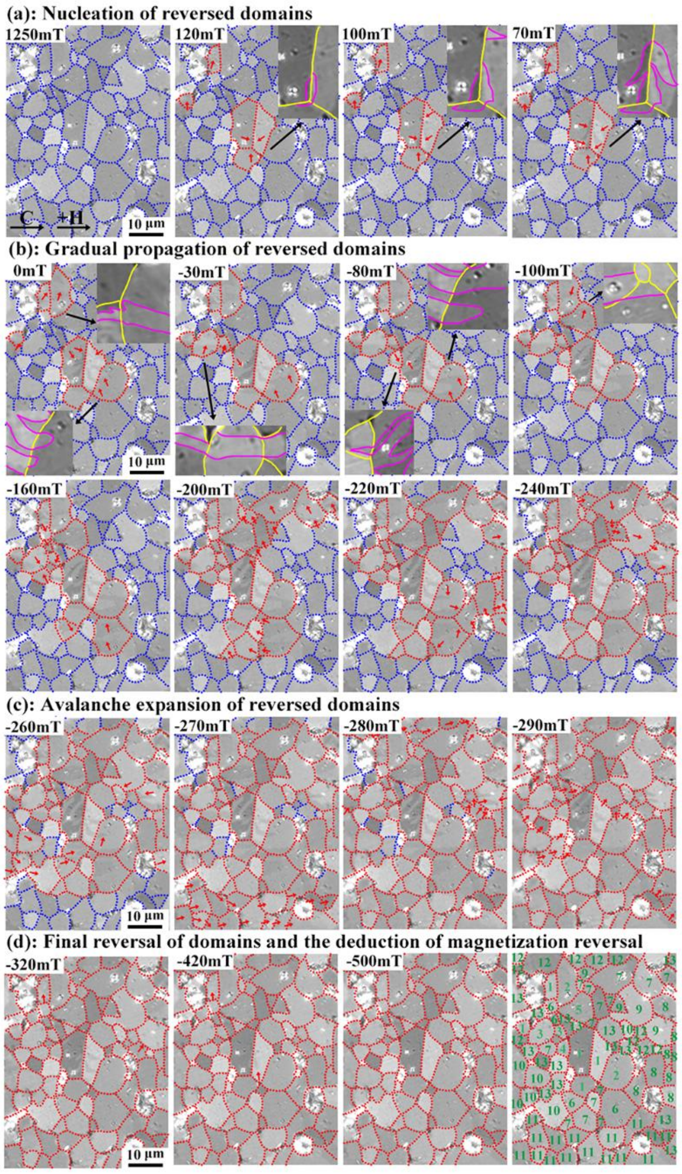

Figure S5. ***In-situ*** **magnetization reversal process of** ***MMP*** **magnet with 18 wt. % La-Ce, recorded by magneto-optical Kerr images.** The external magnetic field is applied to the specimen along the *c*-axis (in-plane). Upon decreasing the external field and afterwards increasing the reversal field, the magnetization reversal can be divided into four steps: (a) nucleation of reversed domains near the GBs at 120 mT, and then grow within the grain and thus become more visible at 100 and 70 mT. (b) Gradual propagation of reversed domains with the increasing opposite field (0~-240 mT). Small nuclei with reversal magnetization tend to grow. Once those nucleation arrives at the GBs that adjacent to another grain, the successive

nucleation in the neighboring grain starts. (c) Avalanche expansion of reversed domains. The domain structure is stable below -240 mT; at higher reversal fields, the nucleation and propagation of reversed domains occur abruptly along the GB, leading to a magnetization cascade. (d) Final reversal of domains, and the corresponding map with "1" to "13" labeled in green, referring to the sequence of reversed domains deduced from the whole reversal progress. At -320 mT, all the present grains have reversed domains within. However, two obvious multi-domain grains remain without being totally reversed until -500 mT (exactly labeled as "1" grains, meaning that they firstly embed with the reversed nucleation and finally occupy the most strong pinning force against the motion of reversed domains).

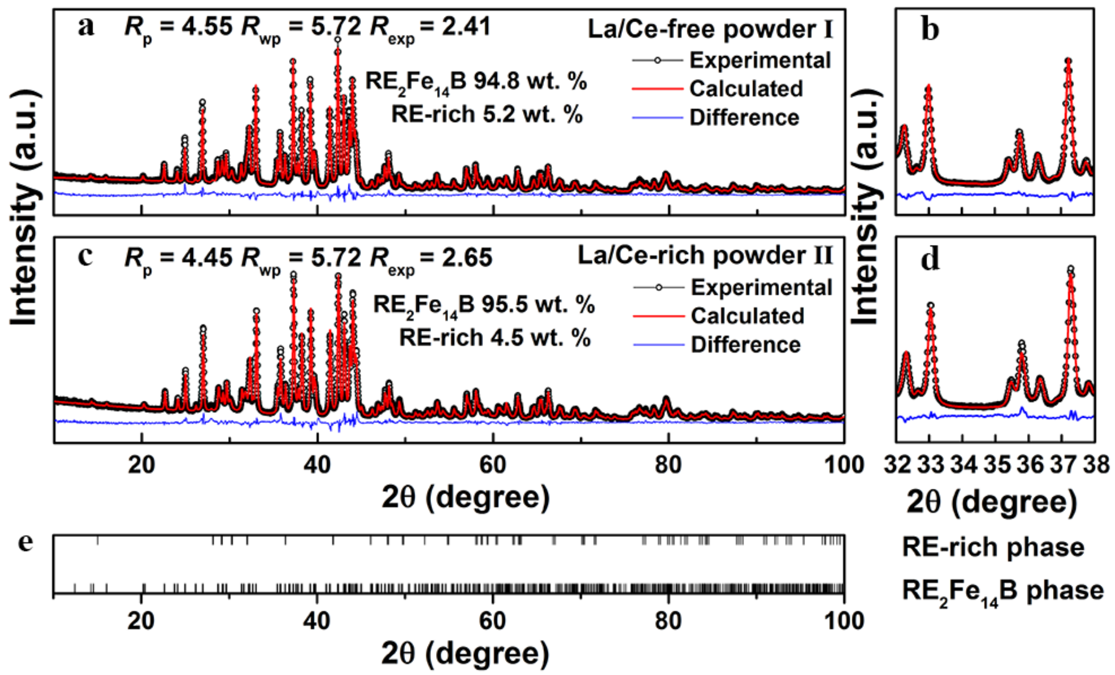

Figure S6. **XRD analysis of the initial La/Ce-free and La/Ce-rich components.** Rietveld refinement of step-scanned XRD patterns for (a) La/Ce-free powder I and (c) La/Ce-rich powder II (La-Ce/TRE = 30 wt. %) at room temperature. Experimental pattern, calculated pattern, and their differences are given in black, red and blue colors, respectively. The difference pattern in each curve indicates a good matching between the calculated and experimental values. Their enlarged XRD patterns of 2θ between 32~38º are shown in (b) and (d), respectively. Bottom ticks in (e) mark the characteristic Bragg positions of $RE_2Fe_{14}B$/RE-rich phase, and serve as a guide to the eye.

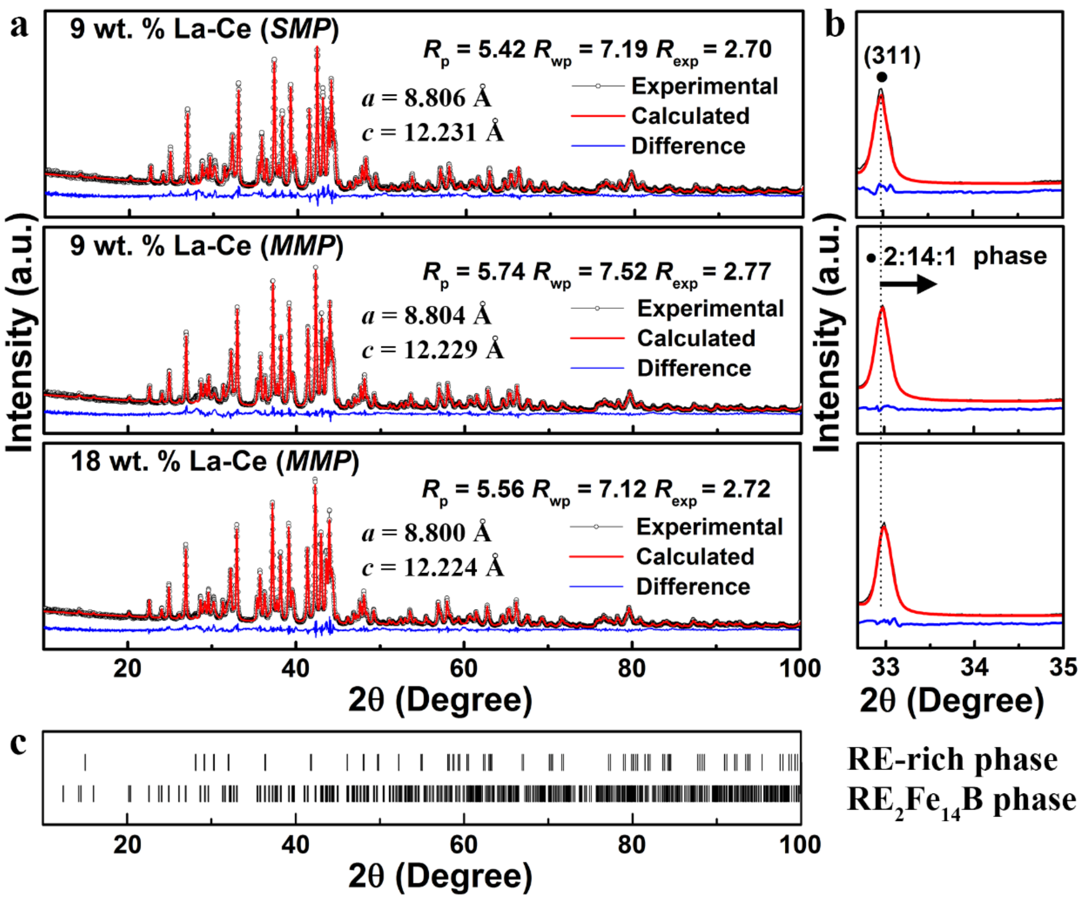

Figure S7. XRD analysis of the *SMP* and *MMP* magnets. (a) Rietveld refinement of step-scanned XRD patterns for La/Ce-containing *SMP* and *MMP* magnets (in powders) at room temperature. Experimental pattern, calculated pattern, and their differences are given in black, red and blue colors, respectively. The difference pattern in each curve indicates a good matching between the calculated and experimental values. Their enlarged XRD patterns of 2θ between 32.8~35º are shown in (b). Bottom ticks in (c) mark the characteristic Bragg positions of $RE_2Fe_{14}B$/RE-rich phase, and serve as a guide to the eye.

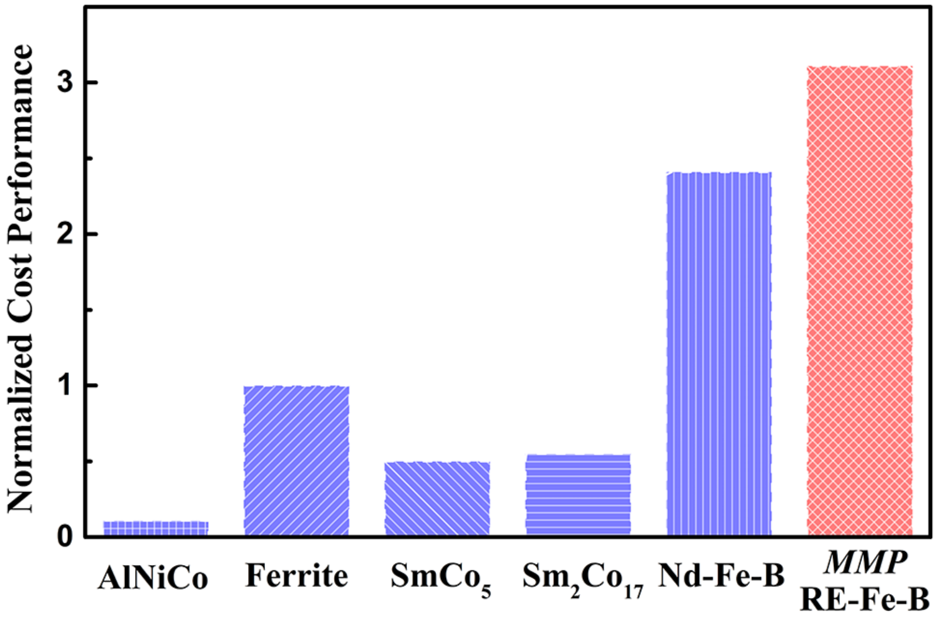

Figure S8. Comparison of cost performance for six classes of technologically important permanent magnetic materials, including AlNiCo, Ferrite, $SmCo_5$, Sm_2Co_{17}, Nd-Fe-B, and newly-developed *MMP* RE-Fe-B magnets. The cost performance for hard ferrites is normalized to 1. The estimation of present *MMP* magnets is based on that the price is 4.4 \$/kg for La-Ce alloy, and 47.5 \$/kg for Pr-Nd alloy (as of December 2015, available at the website[1]).

Explanations for T_C variations:

Curie temperature T_C of the tetragonal $RE_2Fe_{14}B$ compound is determined by three types of coexisting exchange interactions, including RE-RE, RE-Fe, and Fe-Fe. Its expression is as follows[2]:

$$3k_BT_C = Z_{FF}S_F(S_F+1)j_{FF} + Z_{RR}Gj_{RR} + \{[Z_{FF}S_F(S_F+1)j_{FF} - Z_{RR}Gj_{RR}]^2 + \frac{1}{7} Z^2_{RF}S_F(S_F+1)Gj^2_{RF}\}^{1/2} \quad (1)$$

where k_B is the Boltzmann constant, S_F is the Fe spin, and the de Gennes factor $G \equiv (g - 1)^2J(J+1)$. j_{AB} is the exchange interaction energies among A and B spins. For example, j_{FF} refers

to the exchange interaction energies of Fe spins. Z_{AB} is the number of B atom neighbors of atom A. For example, Z_{FF} is the number of Fe atom neighbors.

As the 4f radial matrix elements $<r>_{4f}$ is approximately one order smaller than the RE-RE separations, the direct RE-RE exchange is negligible[2]. Eq. (1) can then be written as:

$$3k_B T_C = Z_{FF} S_F(S_F+1) j_{FF} + \{[Z_{FF} S_F(S_F+1) j_{FF}]^2 + \frac{1}{7} Z^2_{RF} S_F(S_F+1) G j^2_{RF}\}^{1/2} \quad (2)$$

This equation allows one to gain some insight into the variations of T_C for *SMP* and *MMP* magnets. For the specific $RE_2Fe_{14}B$ system, La/Ce substitutions for Pr/Nd do not change the tetragonal structure, thus the coordination numbers Z_{FF} and Z_{RF} remain unchanged. So does the S_F. As a result, we see that in the mean field theory, Curie temperature T_C of 2:14:1 phase increases with larger j_{FF}, j_{RF}, and G. With La/Ce substitution for Pr/Nd in the 2:14:1 lattice, the de Gennes factor G is decreased and the RE-Fe exchange is weakened[3]. The Fe-Fe exchange which accounts for a larger effect on T_C is also reduced[3]. The smaller exchange interaction leads to decreased T_C of La/Ce-containing $RE_2Fe_{14}B$ compounds. In this article, the fact that *MMP* magnets exhibiting higher T_C (300.7 °C) than that (293.9 °C) of *SMP* ones with the same La/Ce concentration of 9 wt. % suggests the strengthened exchange coupling effect of *MMP* magnets (relatively higher j_{FF}/j_{RF}). Though the de Gennes factor G is decreased upon La/Ce substitution, only a slight decline of T_C is observed in 18 wt. % La/Ce-containing *MMP* magnet (299.3 °C) compared to the La/Ce-free starting magnet (302.7 °C), further validating the strong exchange interactions in chemically inhomogeneous *MMP* magnets.

References:

1. Shanghai Metals Market, *Pricing of Metals*. Available at: http://www.metal.com/(2015).

2. Herbst, J. F. $R_2Fe_{14}B$ materials: Intrinsic properties and technological aspects. *Rev. Mod. Phys*. **63**, 819-898 (1991).
3. Alam, A., Khan, M., McCallum, R. W. & Johnson, D. D. Site-preference and valency for rare-earth sites in $(R\text{-}Ce)_2Fe_{14}B$ magnets. *Appl. Phys. Lett*. **102**, 042402 (2013).
